# High prevalence of *Enterocytozoon bieneusi* zoonotic genotype D in captive golden snub-nosed monkey (*Rhinopithecus roxellanae*) in zoos in China

**DOI:** 10.1186/s12917-017-1084-6

**Published:** 2017-06-05

**Authors:** Fuchang Yu, Yayun Wu, Tongyi Li, Jianke Cao, Jiantang Wang, Suhui Hu, Huili Zhu, Sumei Zhang, Rongjun Wang, Changshen Ning, Longxian Zhang

**Affiliations:** 1 College of Animal Science and Veterinary Medicine, Henan Agricultural University, Zhengzhou, 450002 People’s Republic of China; 2 International Joint Research Laboratory for Zoonotic Diseases of Henan, Zhengzhou, 450002 People’s Republic of China; 3 Zhengzhou Zoo, Zhengzhou, 450000 People’s Republic of China; 4 College of Animal Science, Henan Institute of Science and Technology, Xinxiang, 453003 People’s Republic of China

**Keywords:** Microsporidia, Molecular characterization, Phylogeny, Nonhuman primates

## Abstract

**Background:**

*Enterocytozoon bieneusi* is the dominant specie of microsporidia which can infect both anthroponotic and zoonotic species. The golden snub-nosed monkey is an endangered primate which can also infect by *E. bieneusi*. To date, few genetic data on *E. bieneusi* from golden snub-nosed monkeys has been published. Therefore, to clarify the prevalence and genotypes of *E. bieneusi* in captive golden snub-nosed monkeys is necessary to assess the potential for zoonotic transmission.

**Result:**

We examined 160 golden snub-nosed monkeys from six zoos in four cities in China, using PCR and comparative sequence analysis of the ribosomal internal transcribed spacer (ITS). The overall prevalence of *E. bieneusi* was 46.2% (74/160); while the prevalence was 26.7%, 69.1%, 69.4% and 33.3% in Shanghai Zoo, Shanghai Wild Animal Park, Tongling Zoo, and Taiyuan Zoo respectively (*P* = 0.006). A total of seven *E. bieneusi* genotypes were found that included four known (D, J, CHG1, and CHG14) and three new (CM19–CM 21) genotypes. The most common genotype was D (54/74, 73.0%), followed by J (14/74, 18.9%); other genotypes were restricted to one or two samples. Phylogenetic analysis revealed that genotype D belonged to the previously-characterized Group 1, with zoonotic potential; whereas genotypes J, CHG1, CHG14 and CM19–CM 21 clustered in the previously-characterized Group 2, the so-called cattle host specificity group.

**Conclusions:**

The findings of high prevalence of zoonotic *E. bieneusi* genotypes D and J in golden snub-nosed monkeys suggest that golden snub-nosed monkeys may be the reservoir hosts for human microsporidiosis, and vice versa.

**Electronic supplementary material:**

The online version of this article (doi:10.1186/s12917-017-1084-6) contains supplementary material, which is available to authorized users.

## Background

Microsporidia is a relatively diverse clade of unicellular fungi, living as obligate intracellular pathogens, comprising over 1300 species in at least 160 genera [1]. To date, 14 species of microsporidian pathogens have been diagnosed in humans [2], among which *Enterocytozoon bieneusi* is dominant [3]. Manifestations of microsporidiosis caused by *E. bieneusi* include stubborn chronic diarrhea, abdominal pain and weight loss, in immunocompromised people, such as those infected with HIV, organ transplant recipients, cancer patients, and diabetics; while in immunocompetent individuals *E. bieneusi* causes self-limiting diarrhea and malnutrition, and is sometimes asymptomatic [4–6]. *E. bieneusi* has also been detected in variety of animal hosts, including mammals, birds, rodents and reptiles [7, 8].

Since the first identification of *E. bieneusi* in an AIDS patient by Desportes and others in 1985, more than 240 genotypes of the species have been reported from numerous animal hosts, based on sequence analysis of the internal transcribed spacer (ITS) region of the ribosomal RNA (rRNA) gene [8–14]. Phylogenetic analysis of ITS sequences has revealed the presence of eight genotype groups [15]. More than 94% of the identified ITS genotypes of *E. bieneusi* constitute a large group with zoonotic potential, designated as Group 1, while the rest are divided into several host-adapted groups, designated as Groups 2 to 8 [10, 15, 16]. While the molecular phylogeny of these strains is well-studied, the full range of host diversity, including reservoirs and potential zoonotic transmission, remain unresolved issues [17].

The golden snub-nosed monkey is classified as a Category I protected species in China [18]. The species is listed as Category Endangered by the International Union for Conservation of Nature (IUCN), as its numbers have declined by more than 50% in the last 3 generations (approximately 40 years), because of habitat loss. This decline is continuing, though in some areas the populations are declining at a lower rate [19]. In an attempt to improve population numbers and protect the species’ diversity, some golden snub-nosed monkeys have been captured and placed in zoos [20]. Several recent studies have focused on *E. bieneusi* in nonhuman primates (NHPs) in China and in Kenya, with 43 *E. bieneusi* ITS genotypes reported from various NHP species [15, 17, 21]. However, very limited genetic data are available on *E. bieneusi* from golden snub-nosed monkeys. The present study clarifies the prevalence of *E. bieneusi* in captive golden snub-nosed monkeys, as well as assessing the potential for zoonotic transmission.

## Methods

### Study sites and sampling

**Fig. 1:**
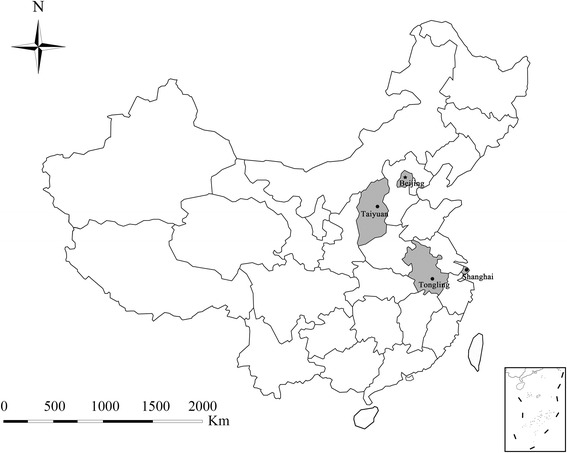
Locations of zoos at which specimens were collected in this study. The figure was generated using the softwares of Chinamap 2.42, Microsoft PowerPoint 2003 and Adobe Photoshop CS6

The study was conducted over a 5-month period (June to November 2015), during that period of time fecal specimens of golden snub-nosed monkeys were collected from Beijing Zoo (116°33′E, 39°94′N), Beijing Wildlife Park (116°33′E, 39°49′N), Shanghai Zoo (121°36′E, 31°19′N), Shanghai Wild Animal Park (121°72′E, 31°05′N), Taiyuan Zoo (112°57′E, 37°91′N) and Tongling Zoo (117°85′E, 30°82′N) (Fig. 1). Some of the monkeys live in single-individual cages; but the majority live as family units in large cages or houses, there are 2 to 4 monkeys in one family unit. The accommodation usually includes play equipment to occupy the animals. To avoid direct contact with visitors, cages are fenced and surrounded by ditches. All the monkeys are usually fed with multi-grain bread twice a day, in the morning and afternoon, with eggs, nuts, seasonal fruits and vegetables as snacks.

A total of 160 fresh fecal specimens were collected from the six zoos in this study (Table 1 and Additional file 1: Table S1 ). All the specimens were collected with the help of local animal attendants, to minimize possible social disruption to the monkeys. Where monkeys were housed individually, fresh fecal deposits were collected in the early morning, as the floors of animal houses were cleaned each evening. For monkeys that were kept in family units in houses during the day, fecal specimens were collected from houses where they spent the night. As the animal handlers described, almost all the monkeys only have one excretion during a whole night, and there are distinct differences among feces from different family members, such as color, smell, size, viscosity, hardness and so on. To avoid duplicate sampling of animals, the fecal deposits were collected for once according to their characteristics. All the specimens were placed into labeled clean zip-lock bags, transported to the Laboratory of Veterinary Parasitology, Henan Agricultural University, in a cooler with ice packs, transferred in water into a 50 ml centrifuge tube, sieved through a 6.5 cm diameter sieve with a pore size of 375 μm (to avoid cross-contamination between samples, the sieve was washed twice with distilled water), and then concentrated by centrifugation. The concentrated fecal specimens were stored in 2.5% potassium dichromate solution at 4 °C prior to DNA extraction.

**Table 1:** Prevalence and ITS genotype distribution of *E. bieneusi* in Golden snub-nosed monkeys in different zoos in China

Study location	No. of specimens	No. (%) of positive specimens	ITS genotypes (no. of specimens)	
			Known	Novel
Beijing Wildlife Park	25	0		
Beijing Zoo	8	0		
Shanghai Zoo	15	4(26.7)	J(2)	CM19(1), CM20(1)
Shanghai Wild Animal Park	55	38(69.1)	D(33), CHG1(1), J(3), CHG14(1)	
Tongling Zoo, Anhui Province	36	25(69.4)	D(21), J(3)	CM21(1)
Taiyuan Zoo, Shanxi Province	21	7(33.3)	J(6)	CM19(1)

### DNA extraction

The stored fecal specimens were washed three times with distilled water after centrifugation, to remove the potassium dichromate. Genomic DNA was extracted using the *E.Z.N.A.R* Stool DNA kit (Omega Biotek Inc., Norcross, USA), following the manufacturer’s protocol. The extracted DNA was stored at −20 °C prior to PCR analysis.

### PCR amplification

The presence of *E. bieneusi* was detected using nested PCR amplification of a 389 bp nucleotide fragment of the ribosomal RNA gene, which included 76 bp of the 3′-end of SSU rRNA gene, the full 243 bp of the ITS region, and 70 bp of the 5′-region of the 5.8S rRNA gene [22]. Outer primers were EBITS3 (5′-GGTCATAGGGATGAAGAG-3′) and EBITS4 (5′-TTCGAGTTCTTTCGCGCTC-3′); EBITS1 (5′-GCTCTGAATATCTATGGCT-3′) and EBITS2.4 (5′-ATCGCCGACGGATCCAAGTG-3′) were used as nested primers for secondary PCR. The cycling parameters were as previously described [23]. Each specimen was analyzed twice, with 1 μl of extracted DNA as template for each PCR, using an Applied Biosystems 2720 Thermal Cycler (Applied Biosystems, Foster City, USA). KOD-Plus enzyme (Toyobo Co. Ltd., Osaka, Japan) was used for PCR amplification. A negative control without DNA was included in all PCRs. Secondary PCR products were examined after 1% agarose gel electrophoresis and staining with GelRed (Biotium Inc., Hayward, CA).

### Nucleotide sequencing and genetic proximity analysis

All positive secondary PCR products were purified using Montage PCR filters (Millipore, Bedford, MA), and sequenced with the secondary PCR primers, by Sinogenomax Biotechnology Co. Ltd. (Beijing, China), using a BigDye Terminator v3.1 cycle sequencing kit (Applied Biosystems) on an ABI 3730 DNA analyzer (Applied Biosystems, Foster City, CA). The accuracy of nucleotide sequences was confirmed by bidirectional sequencing, and by sequencing additional PCR products if necessary.

Sequences were aligned with reference sequences that had been downloaded from GenBank, using Clustal × 2.1 (http://www.clustal.org/). To assess the phylogenetic relationships between ITS genotypes of *E. bieneusi* obtained in the present study and those downloaded from Genbank, a phylogenetic tree was constructed using the Neighbor-Joining(NJ) algorithm, based on a matrix of evolutionary distances calculated using the Kimura 2-parameter model, in MEGA 6.0 (http://www.megasoftware.net/). Confidence in the NJ tree was estimated using a bootstrap analysis with 1000 replicates. Nucleotide sequences obtained in this study have been deposited in GenBank under the accession numbers KU604929–KU604934 and KU685395.

### Statistical analysis

Infection rates were compared using a chi-square test, with differences considered statistically significant at *P* < 0.05.

## Results

### Occurrence and genotypes of *E. bieneusi*

A total of 160 fecal specimens were examined for *E. bieneusi* by PCR, with 74 (46.2%) specimens being positive for *E. bieneusi*. The pathogen was detected in four zoos, including Shanghai Zoo (26.7% of specimens), Shanghai Wild Animal Park (69.1%), Tongling Zoo (69.4%), and Taiyuan Zoo (33.3%), of the six zoos enrolled in this study (Table 1). Differences in prevalence were statistically significant among different zoos (*P* = 0.006). Phylogenetic analysis showed the sequences came from seven distinct *E. bieneusi* genotypes, including four known genotypes (D, J, CHG1, and CHG14) and three new genotypes (named CM19, CM20 and CM21 in this study). As shown in Table 1, genotype D (54/74, 73.0%) was dominant, followed by genotype J (14/74, 18.9%); CM19 was found in two specimens (2/74, 2.7%), while the remaining four genotypes were seen in only one specimen each (1/74, 1.4%).

### Phylogenetic analysis and genetic proximity

**Fig. 2:**
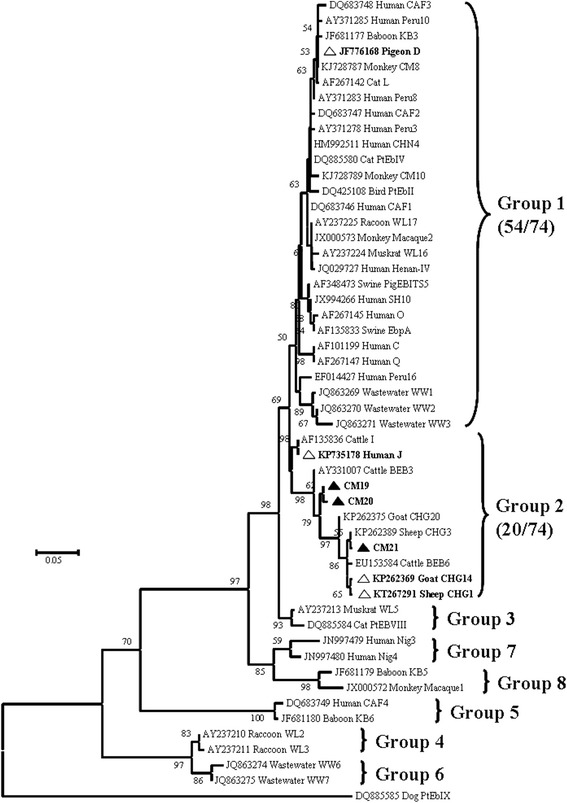
Neighbor-Joining tree of *E. bieneusi* ITS genotypes. The tree was rooted with GenBank sequence DQ885585. Bootstrap values greater than 50% from 1000 replicates are shown on nodes. Each sequence from GenBank is identified by its accession number, host origin, and genotype designation. The group terminology for the clusters is based on previous papers by Thellier and Breton [6], and Karim et al. [15]. Known and new genotypes identified in this study are indicated by open and filled triangles, respectively

Phylogenetic analysis revealed that genotype D belonged to the previously-described Group 1 (Fig. 2). New genotypes CM19 and CM20 differed from genotype CHG20 (KP262375) in Single Nucleotide Polymorphisms (SNPs) at ITS nucleotide positions 177 (an A to G change), 181 (T to C), 261 (G deletion), 268 (G insertion) and 270 (A to G); while CM20 has another SNP at position 177 (A to G). Compared to genotype CHG3 (KP262389), genotype CM21 had one SNP at position 265 (G to T). Genotypes J, CHG1, CHG14 and the three new genotypes (CM19, CM20, and CM21) formed Group 2 in our tree, which has previously been described as the so-called cattle host specificity group (Fig. 2 and Additional file 2: Table S2).

## Discussion

In this study, *E. bieneusi* was found in 46.2% of golden snub-nosed monkeys examined in six zoos in China. The prevalence was higher than that previously reported for golden snub-nosed monkeys in Sichuan (3.5%, 1/29) and Shaanxi (9.5%, 6/63) provinces in China [21, 24]. All of the fecal specimens examined in this study were sampled from six zoos distributed in four locations (Beijing, Shanghai, Anhui Province and Shanxi Province). The infection rates of *E. bieneusi* in monkeys differed among the four zoos that included positive fecal samples. Similar results (11.4%–29.8% prevalence) have previously been reported in different NHP species from various parts of China [15, 17, 21, 24–26]. Perceived rates of infection are influenced by many factors, including the need for laboratory diagnosis, sample sizes, animal management practices, infection intensity of the pathogen, climate and geography. As a parasite with zoonotic potential, *E. bieneusi* has been proved to have the probability to transmit across different NHP species or between animals and animal handlers [27–29]. So the prevalence also maybe influenced by other NHPs living nearby or the animal handlers. As an endangered species which needs more care and protection, the snub-nosed monkeys are usually confined away from other NHP species in all the four zoos that included positive fecal samples. Thus an inference was made that the other NHPs contributed little to the high prevalence in this study. Although the fecal samples of animal handlers were not involved in this study, nevertheless, the transmission probability between monkeys and animal handlers is undeniable.

The zoonotic genotype D, which was the commonest in this study, has been observed frequently in other NHPs, including orangutan, gibbon, macaque and baboon [15, 17, 21, 24–26]. Genotype D has also been identified as the most prevalent genotype in humans, in human wastewater, and in many other animal species (cattle, pigs, sheep, dogs, cats, horses, beavers, otters, muskrats, raccoons, pigeons, falcons and foxes) worldwide [3, 8, 17, 29–34]. For example, a study examining 33 stool samples from human immunodeficiency virus (HIV)-infected adult patients in Thailand showed that genotype D was the most common, found in 36.4% (12 of 33) of the samples [29]. In a recent study, a total of 23 ITS genotypes were obtained from wastewater in Shanghai, Nanjing, Wuhan and Qingdao cities, with genotype D being the most prevalent, found in 279 of 338 (82.5%) positive samples [34]. These results demonstrate that interspecies transmission of genotype D poses a zoonotic risk, as well as being of public health significance already within the human population. The second commonest genotype found in this study, J, is most often found in cattle, though it has been reported as being in humans in Jilin province, China [35]. We have also found this genotype in patients from Henan Province, China (unpublished data). These findings suggest that genotype J may be transmissible from golden snub-nosed monkeys to humans, or vice versa.

The three new genotypes (CM19–CM21), CHG1 and CHG14 were all clustered in the previously-described Group 2, along with the zoonotic genotype J. Although these genotypes fall into a group that has previously been considered cattle-specific, genotype J clearly has the ability to infect humans. Thus, these three new genotypes belonging to Group 2 may also have the ability to cause human microsporidiosis.

## Conclusion

Our study demonstrates a high prevalence of *E. bieneusi* genotypes D and J in golden snub-nosed monkey kept in zoos in China. As these genotypes are common to humans and animals, golden snub-nosed monkeys may serve as potential reservoir hosts for zoonotic *E. bieneusi* genotypes.

## Additional files

Additional file 1: Table S1. Distribution of positive samples in different zoo. (XLS 29 kb)

Additional file 2: Table S2. Nucleotide substitutions among all sequences found in this study versus the reported genotype J (KU557671). (XLS 49 kb)

## Abbreviations

HIV: Human immunodeficiency virus
ITS: Internal transcribed spacer
IUCN: The International Union for Conservation of Nature
NHPs: Nonhuman primates
rRNA: Ribosomal RNA
